# Effectiveness of Wellbeing Intervention for Chronic Kidney Disease (WICKD): results of a randomised controlled trial

**DOI:** 10.1186/s12882-021-02344-8

**Published:** 2021-04-19

**Authors:** Kylie M. Dingwall, Michelle Sweet, Alan Cass, Jaquelyne T. Hughes, David Kavanagh, Kirsten Howard, Federica Barzi, Sarah Brown, Cherian Sajiv, Sandawana W. Majoni, Tricia Nagel

**Affiliations:** 1 Menzies School of Health Research, Charles Darwin University, Alice Springs, NT 0870 Australia; 2 Menzies School of Health Research, Charles Darwin University, Darwin, NT 0811 Australia; 3 Division of Medicine, Royal Darwin Hospital, Darwin, NT 0811 Australia; 4 Department of Nephrology, Royal Darwin Hospital, Northern Territory Department of Health, Darwin, NT 0810 Australia; 5 Centre for Children’s Health Research and School of Psychology & Counselling, Faculty of Health, Queensland University of Technology (QUT), Brisbane, QLD 4101 Australia; 6 Sydney School of Public Health, Faculty of Medicine and Health, University of Sydney, Sydney, NSW 2006 Australia; 7 Western Desert Nganampa Walytija Palyantjaku Tjutaku, Alice Springs, NT 0870 Australia; 8 Central Australian Renal Services, Alice Springs Hospital, Northern Territory Department of Health, Alice Springs, NT 0870 Australia; 9 Northern Territory Medical Program, Flinders University, Darwin, NT 0815 Australia

**Keywords:** Renal, E-mental health, Indigenous, Wellbeing, Kidney disease

## Abstract

**Background:**

End stage kidney disease (ESKD) is associated with many losses, subsequently impacting mental wellbeing. Few studies have investigated the efficacy of psychosocial interventions for people with ESKD and none exist for Indigenous people, a population in which the ESKD burden is especially high.

**Methods:**

This three-arm, waitlist, single-blind randomised controlled trial examined efficacy of the Stay Strong App in improving psychological distress (Kessler distress scale; K10), depressive symptoms (adapted Patient Health Questionnaire; PHQ-9), quality of life (EuroQoL; EQ. 5D) and dialysis adherence among Indigenous Australians undergoing haemodialysis in central and northern Australia (Alice Springs and Darwin), with follow up over two 3-month periods. Effects of immediate AIMhi Stay Strong App treatment were compared with those from a contact control app (The Hep B Story) and treatment as usual (TAU). Control conditions received the Stay Strong intervention after 3 months.

**Results:**

Primary analyses of the full sample (*N* = 156) showed statistically significant decreases in K10 and PHQ-9 scores at 3 months for the Hep B Story but not for the Stay Strong app or TAU. Restricting the sample to those with moderate to severe symptoms of distress or depression (K10 > =25 or PHQ-9 > =10) showed significant decreases in K10 and PHQ-9 scores for both Stay Strong and Hep B Story. No significant differences were observed for the EQ-5D or dialysis attendance.

**Conclusions:**

Findings suggest that talking to people about their wellbeing and providing information relevant to kidney health using culturally adapted, locally relevant apps improve the wellbeing of people on dialysis. Further research is required to replicate these findings and identify active intervention components.

**Trial registration:**

ACTRN12617000249358; 17/02/2017.

## Background

End-stage kidney disease (ESKD) is the most severe form of chronic kidney disease (CKD) and requires a transplant or dialysis for survival. In Australia, rates of ESKD are higher among the elderly, Indigenous Australians and people living in remote and socioeconomically disadvantaged areas [1–3]. Australia’s Northern Territory is a large geographical area consisting of a relatively small population of non-Indigenous people living mostly in urban centres along with smaller Indigenous groups living mostly in remote settings distant from centralised services [4]. The majority of Indigenous ESKD patients receive ongoing haemodialysis treatment in a hospital satellite unit setting. For Indigenous patients in the NT, access to haemodialysis usually requires relocation to urban centres adding to the already high emotional burden associated with ESKD.

Indigenous patients are known to demonstrate extraordinary ‘tenacity and ‘resilience’ in the face of ESKD and its impacts [5]. Nevertheless, depression can be common in people undergoing dialysis (25% show depressive symptoms when assessed by clinical interview, 40% when assessed by self-report measures) [6] with depressive symptoms considered a risk factor for poor outcome [7].

Psychosocial interventions for ESKD may prevent or minimise impact of debilitating mental disorders and wellbeing concerns, however well designed intervention studies are lacking for both mainstream and Indigenous CKD populations [7, 8]. Furthermore, there is a significant lack of rigorous effectiveness trials for mental health interventions in an Indigenous context generally [9].

Recent technological advancements and government initiatives have rendered digital mental health (dMH) interventions more popular due to their accessibility and increasing evidence [10–12]. Culturally responsive, strengths-based, early-intervention mental health treatment programs are considered most appropriate to influence the high rates of psychological distress and suicide experienced by Indigenous Australians [13]. The Aboriginal and Islander Mental Health Initiative (AIMhi) Stay Strong care plan is a culturally-adapted, well-researched, effective engagement and low-intensity treatment strategy for improving Indigenous SEWB [14–17]. It adopts an empowering, person-centred, holistic and strengths based approach which acknowledges and promotes Indigenous cultural and family values and client self-management [18] and has recently been translated into a digital (tablet) format (the AIMhi Stay Strong App) and adapted for the Indigenous ESKD setting [19, 20].

This study aimed to determine whether the AIMhi Stay Strong App improves mental health and wellbeing for Indigenous people receiving haemodialysis, relative to delayed-treatment control groups at 3 months, and whether benefits are maintained at 6 months post-recruitment.

## Methods

### Hypotheses

We hypothesised that the AIMhi Stay Strong App would be superior to both a contact control (another app - Hep B Story) [21] and usual care, in reducing psychological distress and depressive symptoms, and improving quality of life, and dialysis adherence at 3 months. We also expected that delivering the Stay Strong app to control groups after the 3-month assessment would result in those groups showing improvements in these outcomes between 3 and 6 months.

### Study design and participants

The study methods have been detailed previously [17] and are summarised below.

**Fig. 1:**
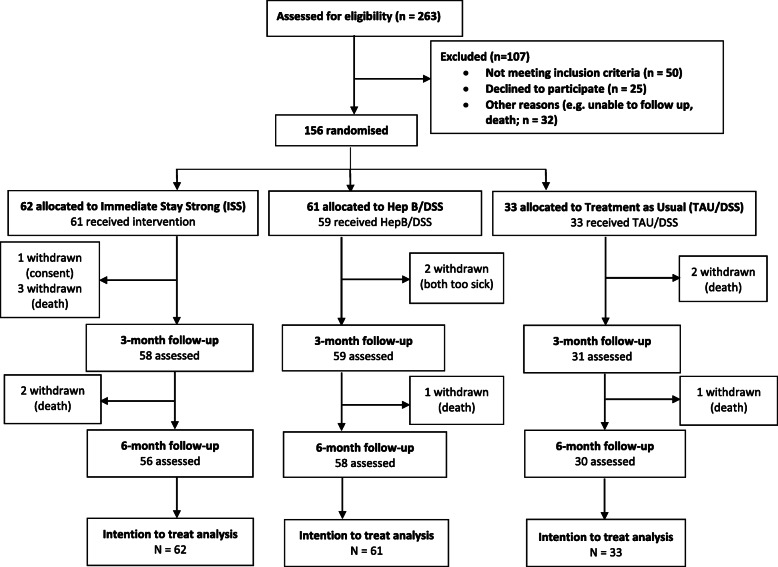
CONSORT diagram of participant flow through the study

This was a three-arm, waitlist, single-blind randomised controlled trial with 2:2:1 allocation ratio testing the efficacy of the Stay Strong App intervention in improving wellbeing among Indigenous clients undergoing haemodialysis for ESKD in Alice Springs and Darwin. Assessments occurred at Baseline, 3 and 6 months (see Fig. 1 for CONSORT diagram). The three treatment conditions were: 1) Immediate treatment with the Stay Strong App (“ISS”), 2) Contact control/Delayed Stay Strong treatment (i.e. patients are engaged with the researcher for a similar time using the Hep B Story app then received the Stay Strong App at 3 months; “HepB/DSS”), and 3) Treatment as usual/Delayed Stay Strong treatment after 3 months (“TAU/DSS”; see Fig. 1).

Inclusion criteria were Aboriginal and Torres Strait Islander and aged ≥18 years, receiving maintenance haemodialysis in Alice Springs or Darwin for more than 6 months. Exclusion Criteria were aged < 18 years, guardianship order in place, or inability to provide informed consent (e.g. because of cognitive or visual impairment). No major changes to the study protocol occurred after trial commencement.

### Consent, ethics and culturally appropriate approach

This study was performed in accordance with the Declaration of Helsinki. Approvals were granted by the Central Australian Human Research Ethics Committee (CAHREC No: HREC-16-406) and the Human Research Ethics Committee (HREC) for the NT Department of Health and Menzies School of Health Research (HREC-16-2599), including an Aboriginal subcommittee. Fully informed oral consent was obtained from all participants using pictorial information sheets and flipcharts in plain English with Aboriginal language versions available. Demographic information and outcome measures were collected using a tablet device including pictorial prompts and Aboriginal language recordings (choice of 11 NT languages). Interpreters were utilised where necessary.

### Interventions

In addition to their allocated treatment, all participants received usual care from their renal service. Usual care was carried out according to the norms prevailing in the renal service, informed by the needs of the client. Interventions are described in detail elsewhere and outlined below [17].

#### Immediate AIMhi stay strong app treatment (ISS)

##### Baseline

Participants randomised to ISS completed a ~ 20-min interview using the AIMhi Stay Strong App at baseline, with a second ~ 20-min session using the App within 2–4 weeks. Session 1 explored family, strengths, worries and goal setting. Session 2 reviewed information entered previously, refined the goals and addressed any barriers to goal attainment, setting new goals as appropriate. Participants received a text message or phone call 1 week following the initial treatment reminding them of their goals and steps for making changes.

##### Three Months

Participants received a further two sessions using the AIMhi Stay Strong app following the 3-month follow-up assessment. The two 20-min sessions occurred 2–4 weeks apart following a similar format to the earlier sessions. A text message or phone call was sent 1 week following the initial treatment to remind participants of their goals and steps for making change and the time for the next session.

#### Hep B story contact control/delayed stay strong treatment (HepB/DSS)

##### Baseline

Participants randomised to HepB/DSS received 20 min of contact with the researcher using a culturally appropriate health app (i.e. The Hep B Story) at baseline, with a further 20-min session using the same app after 2–4 weeks. This ensured that each group received the same contact time and controlled for use of an app to structure the control session, as well as assisting with participant blinding. In Session 1 the participant interacted with the Hep B Story app with support from the researcher, focusing on app navigation and content. A ‘goal’ to talk to someone else in their family about the app content before the next session was set. Participants received a pictorial summary (utilising similar colours and images to the intervention summary). Session 2 reviewed the information discussed in Session 1. Participants received a text message or phone call reminding them of their goal (to talk with someone about the information) in the intervening weeks.

##### Three months

Participants received a 20-min interview using the AIMhi Stay Strong App (on a tablet device) following their 3-month assessment, with one further 20-min session using the App within 2–4 weeks, following the format of the sessions received by the ISS group at baseline.

#### Treatment as usual/delayed stay strong treatment (TAU/DSS)

##### Baseline

Participants who are randomised to TAU/DSS only received the questionnaires and no other researcher intervention at baseline.

##### Three months

After the 3-month assessment, participants received the AIMhi Stay Strong app intervention, using the same procedures as the HepB/DSS group.

#### Fidelity of the intervention

The interventions were delivered by trained researchers with reference to the AIMhi Stay Strong Planning Brief Treatment Manual [22]. Reviews of App data and ongoing booster sessions were used by the research team to provide regular feedback to redirect and adjust their mode of delivery as needed [17]. We defined an acceptable level of fidelity as two sessions of treatment delivered, one of which was at least 15 min. This applied to both the contact control (Hep B app) and intervention (Stay Strong app).

### Outcomes

All participants were assessed at the beginning of the study (T0), after 3 months (T3) and again after 6 months (T6) with the following instruments: (1) the Kessler Distress Scale (K10, 2) the adapted Patient Health Questionnaire (PHQ-9); and the EuroQol (EQ-5D) 5 level. The K10 is a 10-item measure of psychological distress that is sensitive to symptoms of both anxiety and depression [23]. Responses are on a 5-point Likert scale. The PHQ-9 is a 9-item measure of depressive symptoms, scored on a 4-point Likert scale and has been adapted and tested for Australian Aboriginal people [24]. EQ-5D is a self-report measure of quality of life in 5 domains (mobility, self-care, usual activities, pain and discomfort, anxiety and depression). Respondents rate their health today on 5 levels of severity. It also includes a visual analogue scale which is used as a quantitative measure of overall health status. These assessments and their justification are described in detail elsewhere [17].

### Sample size and power

The sample size calculation aimed to detect a minimum difference of 5 points on the K10 (with 90% power and an alpha of .05) leading to a sample size of 62, 62 and 32 for the ISS, HepB/DSS and TAU/DSS groups respectively. Based on previous data we considered a difference between the group mean scores of 5 to be clinically significant. This calculation allowed for 10% attrition.

### Randomisation

Participants were randomised using a block sequential random number sequence (block sizes of 2 & 3) and an envelope system of randomisation, stratified by site, level of psychological distress (high or low i.e. < or > 25) and access to respite dialysis in home community as a person’s ability to return home to family (for visits) may influence wellbeing. An independent statistician created the allocation schedule with a computerized random number generator and investigators were blind to this schedule. Following baseline assessment, research assistants selected the next envelope in the sequence based on the participants site, level of distress and access to dialysis at home. Participants were allocated to ISS, HepB/DSS or TAU/DSS at a ratio of 2:2:1. The allocation was concealed to participants.

### Blinding

Participants and outcome assessors were blinded to treatment condition. Research assistants who delivered the intervention were restricted from conducting follow up assessments with those participants to maintain blinding. The contact control involved an intervention of similar length of time and utilised an app presented on a tablet device to minimise client awareness of their treatment group assignment.

### Statistical analyses

The primary analyses were conducted according to the intention-to-treat principle.

Descriptive statistics of baseline characteristics are provided by randomisation group. Continuous variables are summarised with mean and standard deviation (SD) when appropriate, or median and interquartile range (IQR) when not normally distributed, categorical variables were summarized with frequency and percentage.

Linear mixed models were used to estimate, for each of the K10, the EQ-5D and PHQ-9 score, the change between: (i) baseline assessment and 3-month assessment (ii) baseline and 6-month assessment (iii) 3-month and 6-month assessment. The changes described above were also compared between groups (ISS vs HepB/DSS; ISS vs TAU/DSS; HepB/DSS vs TAU/DSS) with mixed models that included a categorical variable for the three intervention arms, for each time point and an interaction term between arm and time. The use of mixed models has the advantage of allowing the modelling of the correlation between repeats of outcome recorded within the same participant and within site. Sensitivity analyses including baseline scores were included in the models when descriptive analyses showed important between intervention arm differences for baseline scores. The above described analyses were carried out for subgroups defined by baseline score severity (below or above 25 for K10 and below and above 10 for the PHQ-9), by region (Top End and Central Australia), access to home community dialysis or not.

Zero inflated Poisson models were used to calculate and compare between allocation arms, mean and 95% confidence intervals of numbers of missing dialysis sessions during the baseline to 3-month period, and the 4–6-month period.

## Results

### Participants

Sixty-two participants were allocated to receive ISS, 61 to HepB/DSS, and 33 to TAU/DSS. Data collection occurred between February 2017 and March 2019. Participant flow is described in Fig. 1. Baseline descriptive statistics are presented in Table 1. Nine participants died during the study, for reasons unrelated to the trial and consistent with the high mortality experienced generally amongst the dialysis population, and two were withdrawn due to being too ill to participate (see Fig. 1). Follow up at 3 months as achieved for 93.4% of the ISS group, 96.7% of the HepB/DSS group and 93.9% of the TAU/DSS group. At 6 months, follow up was achieved for 90.3, 95.1 and 90.9% respectively. Overall attrition was 7.7%. The trial ended when the proposed sample size was achieved.

**Table 1:** Summary of baseline characteristics by intervention group

		ISS ***N*** = 62	HepB/DSS ***N*** = 61	TAU/DSS ***N*** = 33	Total ***N*** = 156
Gender:	Male	16 (25.8)	19 (31.2)	9 (27.3)	44 (28.2)
	Female	46 (74.2)	42 (68.9)	24 (72.7)	112 (71.8)
Age at randomisation:	(years)	55 (10.6)	53.9 (8.7)	57.0 (8.2)	55 (9.4)
Years since initiation of dialysis	Median [IQR]	3.4 [2.1–8.5]	2.8 [1.6–5.2]	3.6 [2.4–5.7]	3.1 [2.0–5.7]
English as 1st language	N (%)	10 (16%)	11 (18%)	7 (21%)	28 (18%)
Access to dialysis at home community	N (%)	37 (60%)	38 (62%)	22 (67%)	97 (62%)
Top End	N (%)	32 (52%)	33 (54%)	13 (39%)	78 (50%)
Central Australia	N (%)	30 (48%)	28 (46%)	20 (61%)	78 (50%)
K-10	Mean (SD)	21.4 (7.4)	23.6 (8.6)	22.3 (8.8)	22.4 (8.2)
PHQ-9	Mean (SD)	8.4 (5.2)	9.1 (5.7)	7.9 (4.4)	8.6 (5.2)
EQ-5D	Mean (SD)	74.5 (21.7)	70.1 (18.2)	77.9 (17.2)	73.5 (19.6)

### Fidelity

Table 2 reports number of missed sessions and average minutes for each session by treatment group. For the ISS arm, the number who received the intervention with an acceptable level of fidelity (2 sessions, one of which was greater than or equal to 15 min) was 48/61 (79%) the early treatment period and 37/56 (66%) at the delayed treatment period. For the Hep B/DSS group, the number who received the intervention with an acceptable level of fidelity (described above) was 41/59 (70%) for the early (i.e. Hep B) treatment period and 45/58 (78%) for the delayed (i.e. Stay Strong) treatment period. For TAU/DSS, 18/30 (60%) received Stay Strong with an acceptable level of fidelity at the delayed treatment period.

**Table 2:** Fidelity data by intervention group

	Immediate Stay Strong (ISS)		HepB/DSS		TAU/DSS	
	Number missed	Average minutes	Number missed	Average minutes	Number missed	Average minutes
Immediate Treatment						
**session 1**	1/62	24.09	3/61	17.55	–	–
**session 2**	7/62	19.04	13/61	15.15	–	–
Delayed Treatment – Stay Strong						
**session 1**	5/62	22.34	2/61	24.86	5/33	24.57
**session 2**	14/62	16.30	9/61	14.12	7/33	15.31

### Changes in K10 over time and between arms

**Fig. 2:**
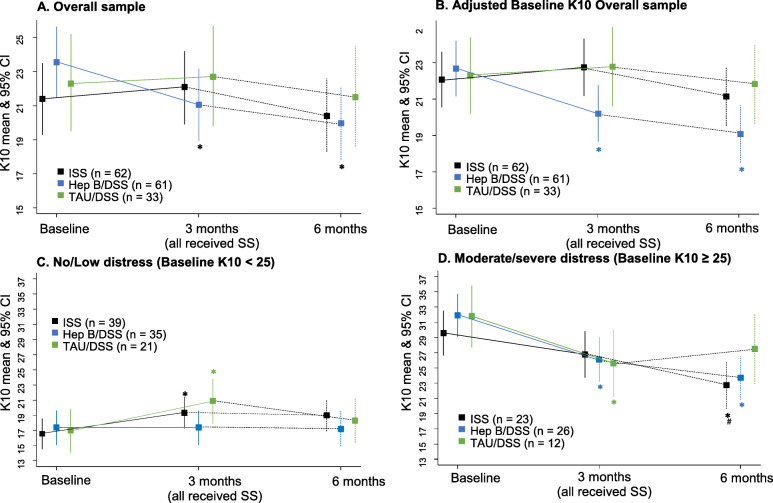
K10 mean and standard deviation over the three time points by allocation group. The symbol * indicate significant change from baseline at *p* < 0.05. The symbol # indicates significant change from 3 to 6 months at *p* < 0.05

Participants in the HepB/DSS group showed a statistically significant decrease (2.5 points) in K10 score from baseline to 3-months (95% CI: 0.5–4.6; *p* = 0.02) and more so from baseline to 6 months with a decline of 3.5 points (95% CI: 1.5–5.7; *p* = 0.001) however these changes were not clinically significant (i.e. > = 5points). Significant reductions in K10 scores in the ISS and TAU/DSS arms were not evident. There was no substantial difference in scores between groups over time, however, when compared to ISS, HepB/DSS had a marginally greater decrease from baseline to 3-months [3.2 (0.3–6.1); *p* = 0.03]. (Fig. 2; Table 3, 4).

**Table 3:** Mean (and 95%CI) K10, PHQ-9, and EQ-5D scores at Baseline, 3-months and 6-months for full sample

Measure	Timepoint	ISS		HepB/DSS		TAU/DSS	
		n	Mean (95% CI)	n	Mean (95% CI)	n	Mean (95% CI)
K10	Baseline	62	21.4 (19.3–23.5)	61	23.6 (21.5–25.7)	33	22.3 (19.5–25.2)
	3-months	58	22.1 (19.9–24.2)	59	21.1 (18.9–23.2)	30	22.7 (19.8–25.7)
	6-months	56	20.4 (18.3–22.6)	57	20.0 (17.8–22.1)	30	21.5 (18.6–24.5)
PHQ-9	Baseline	62	8.4 (7.1–9.7)	61	9.1 (7.7–10.4)	33	7.9 (6.1–9.7)
	3-months	58	8.2 (6.8–9.6)	59	7.5 (6.1–8.9)	30	8.8 (6.9–10.6)
	6-months	56	7.7 (6.3–9.0)	57	7.6 (6.3–9.0)	30	8.2 (6.3–10.1)
EQ-5D	Baseline	62	0.78 (0.72–0.84)	31	0.79 (0.72–0.85)	33	0.83 (0.74–0.91)
	3 months	58	0.77 (0.70–0.83)	59	0.84 (0.78–0.90)	30	0.79 (0.71–0.88)
	6 months	56	0.82 (0.75–0.88)	57	0.83 (0.76–0.89)	30	0.82 (0.73–0.91)

**Table 4:** Mean difference (and 95% CI) from Baseline to 3 months (T0-T3), Baseline to 6 months (T6-T0) and 3 months to 6 months (T6-T3) within allocated group and between groups for full sample

Measure	Timepoint	ISS	HepB/DSS	TAU/DSS	ISS vs HepB/DSS	ISS vs TAU/DSS	HepB/DSS vs TAU/DSS
K10	T3-T0	0.7 (−1.4,2.8)	−2.5 (− 4.6,-0.5)*	0.4 (− 2.4,3.3)	3.2 (0.3,6.1)*	0.3 (− 3.2,3.8)	− 2.9 (− 6.4,0.6)
	T6-T0	− 1.0 (− 3.0,1.1)	− 3.6 (− 5.7,-1.5)**	−0.8 (− 3.6,2.1)	2.6 (− 0.3,5.6)	− 0.2 (− 3.7,3.4)	−2.8 (− 3.3,0.7)
	T6-T3	− 1.6 (− 3.8,0.5)	− 1.1 (− 3.2,1.0)	− 1.2 (− 4.1,1.7)	−0.6 (− 3.5,2.4)	− 0.4 (− 4.0,3.1)	0.1 (− 3.5,3.7)
PHQ-9	T3-T0	− 0.02 (− 3.2,0.4)	−1.6 (− 2.9,-0.3)*	0.8 (− 1.0,2.7)	1.4 (− 0.4,3.2)	−1.0 (− 3.3,1.2)	− 2.4 (− 4.7,-0.2)
	T6-T0	−0.7 (−2.1,0.6)	− 1.4 (− 2.8,-0.1)*	0.3 (− 1.6,2.1)	0.7 (− 1.2,2.6)	−1.0 (− 3.2,1.3)	−1.7 (− 3.9,0.5)
	T6-T3	−0.5 (− 1.9,0.8)	0.1 (− 1.2,1.5)	−0.6 (− 2.4,1.2)	−0.7 (− 2.6,1.2)	0.1 (− 2.2,2.3)	0.7 (− 1.5,3.0)

*difference is significant at *p*< 0.05

** difference is significant at *p*< 0.001

**Fig. 3:**
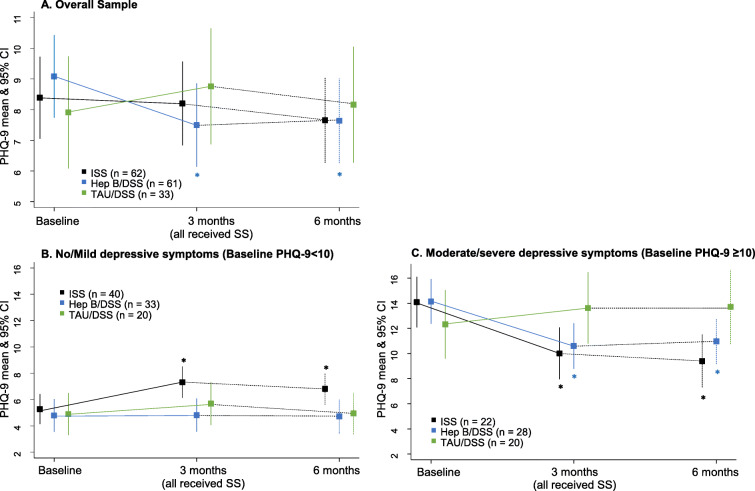
PHQ-9 mean and standard deviation over the three time points by allocation group. The symbol * indicates significant change from baseline at *p* < 0.05

The subgroup analyses by baseline severity indicated a statistically and clinically significant effect of the ISS intervention at 6 months for those with a baseline K10 greater than or equal to 25. Similar results to both the ISS results and the primary analysis were found for the HepB/DSS group, with changes also attaining clinical significance (see Tables 5 & 6). While there was no significant difference in mean changes between groups, there was a trend for ISS to show a marginally greater decrease in K10 scores than TAU/DSS from 3 to 6 months [6.0 (0.1–12.0) *p* = 0.05] (see Fig. 3).

**Table 5:** Mean (and 95%CI) K10, and PHQ-9 scores at Baseline and 3-months and 6-months for sub-analyses by symptom severity

Measure	Timepoint	ISS		HepB/DSS		TAU/DSS	
		n	Mean & 95% CI	n	Mean & 95% CI	n	Mean & 95% CI
K10 > =25	Baseline	23	29.6 (26.6–32.5)	26	31.9 (29.1–34.7)	12	31.8 (27.7–35.8)
	3-months	21	26.8 (23.7–29.8)	24	26.1 (23.7–29.0)	10	25.6 (21.2–30.0)
	6-months	20	22.7 (19.6–25.9)	24	23.7 (20.8–26.5)	9	27.5 (22.9–32.1)
K10 < 25	Baseline	39	16.6 (14.5–18.6)	35	17.3 (15.1–19.6)	21	17.0 (14.1–19.9)
	3-months	37	19.3 (17.3–21.4)	35	17.3 (15.1–19.6)	20	20.9 (17.9–23.8)
	6-months	36	19.0 (16.9–21.1)	33	17.2 (14.9–19.5)	21	18.3 (15.4–21.2)
PHQ-9 > =10	Baseline	22	14.1 (12.1–16.1)	28	14.1 (12.4–15.9)	13	12.3 (9.6–15.1)
	3-months	21	10.0 (8.0–12.1)	27	10.6 (8.8–12.4)	11	13.6 (10.8–16.5)
	6-months	20	9.4 (7.3–11.5)	27	11.0 (9.1–12.8)	10	13.7 (10.8–16.7)
PHQ-9 < 10	Baseline	40	5.3 (4.1–6.4)	33	4.8 (3.5–6.0)	20	4.9 (3.3–6.5)
	3-months	37	7.3 (6.1–8.5)	32	4.8 (3.6–6.1)	19	5.7 (4.1–7.3)
	6-months	36	6.8 (5.6–8.0)	30	4.7 (3.4–6.0)	20	5.0 (3.4–6.5)

**Table 6:** Mean difference (and 95% CI) from Baseline to 3 months (T0-T3), Baseline to 6 months (T6-T0) and 3 months to 6 months (T6-T3) within allocated group and between groups by symptom severity

Measure	Timepoint	ISS	HepB/DSS	TAU/DSS	ISS vs HepB/DSS	ISS vs TAU/DSS	HepB/DSS vs TAU/DSS
K10 > =25	T3-T0	−2.8 (−6.1, 0.5)	−5.8 (−8.9, −2.7)**	−6.2 (−10.9, −1.5)*	3.0 (−1.5, 7.5)	3.4 (− 2.4, 9.2)	0.4 (− 5.3, 6.0)
	T6-T0	−6.8 (− 10.2, −3.5)**	− 8.2 (− 11.3, − 5.1)**	−4.2 (−9.1, 0.7)	1.4 (− 3.2, 5.9)	−2.6 (− 8.5, 3.3)	− 4.0 (− 9.8, 1.8)
	T6-T3	−4.0 (−7.4, −0.7)*	−2.4 (− 5.5, 0.7)	2.0 (− 3.1, 7.0)	− 1.6 (− 6.2, 3.0)	−6.0 (− 12.0, 0.1)~	−4.4 (− 10.3, 1.5)
K10 < 25	T3-T0	2.8 (0.5, 5.1)*	0.0 (− 2.4, 2.4)	3.9 (0.8, 7.0)*	2.8 (−0.5, 6.1)	− 1.2 (− 5.0, 2.7)	− 3.9 (− 7.8, 0.01)
	T6-T0	2.4 (0.1, 4.8)*	−0.1 (− 2.6, 2.3)	1.3 (− 1.7, 4.4)	2.6 (− 0.8, 5.9)	1.1 (−2.7, 4.9)	− 1.5 (− 5.4, 2.4)
	T6-T3	−0.3 (− 2.7, 2.0)	−0.1 (− 2.6, 2.3)	−2.6 (− 5.7, 0.5)	−0.2 (− 3.6, 3.2)	2.3 (− 1.6, 6.2)	2.5 (− 1.5, 6.4)
PHQ-9 > =10	T3-T0	−4.1 (− 6.4, − 1.8)**	−3.5 (− 5.6, − 1.5)*	1.3 (− 1.9, 4.4)	−0.5 (− 3.6, 2.5)	−5.4 (− 9.3, − 1.5)*	− 4.8 (− 8.6, − 1.1)*
	T6-T0	−4.7 (− 7.0, − 2.4)**	−3.2 (− 2.2, − 1.2)*	1.4 (−1.9, 4.6)	− 1.5 (− 4.6, 1.6)	−6.1 (− 10.1, − 2.1)*	−4.6 (− 8.4, − 0.7)*
	T6-T3	−0.6 (− 2.9, 1.7)	0.4 (−1.7, 2.4)	0.1 (−3.2, 3.4)	− 1.0 (− 4.1, 2.1)	−0.7 (− 4.7, 3.3)	0.3 (− 3.6, 4.1)
PHQ-9 < 10	T3-T0	2.0 (0.6, 3.4)*	0.0 (− 1.5, 1.5)	0.8 (− 1.4, 2.7)	2.0 (0.0, 4.1)	1.2 (− 1.1, 3.6)	−0.8 (− 3.2, 1.7)
	T6-T0	1.5 (0.1, 2.9)*	−0.1 (− 1.6, 1.5)	0.0 (− 1.9, 2.0)	1.6 (− 0.5, 3.7)	1.5 (− 0.9, 3.8)	−0.1 (− 2.6, 2.3)
	T6-T3	−0.5 (− 1.9, 0.9)	− 0.1 (− 1.6, 1.4)	−0.7 (− 2.7, 1.2)	−0.4 (− 2.5, 1.7)	0.2 (− 2.2, 2.6)	0.6 (− 1.8, 3.1)

*difference is significant at *p*< 0.05

** difference is significant at *p*< 0.001

No significant decreases in K10 scores were observed in the subgroup analyses for any treatment arm when baseline K10 was less than 25.

There were no substantive changes after adjusting by baseline K10 so unadjusted results are reported.

### Changes in PHQ-9 over time and between arms

PHQ-9 changes were similar to those of K10. Primary analyses indicated, participants in the HepB/DSS group showed a statistically significant (but not clinically significant) decrease (1.6 points) in the total PHQ-9 score from baseline to 3 months (95% CI: 0.3–2.9; *p* = 0.02 which remained at 6 months (*p* = 0.03). Significant reductions in PHQ-9 scores for the ISS and TAU/DSS arms were not evident. There was no substantial difference in PHQ-9 scores between groups over time, however, when compared to TAU/DSS, HepB/DSS had a marginally greater decrease from baseline to 3 months [2.4 (0.2–4.7); *p* = 0.03]. There was no adjustment made for baseline PHQ-9 scores given the similar scores between groups at baseline.

The subgroup analyses by baseline severity indicated an effect of the ISS for those with a baseline PHQ-9 greater than or equal to 10. For this subgroup, there was a statistically significant decrease (4.08 points) in PHQ-9 score from baseline to 3 months which remained at 6 months. While reductions were less than the 5 points required to reach clinical significance, the changes approached clinical significance (4.08 points at 3 months and 4.68 points at 6 months). Similar results to this and the overall were found for the HepB/DSS group with changes failing to reach clinical significance (3.55 and 3.18 at 3 and 6 months respectively). Both ISS and HepB/DSS showed significantly larger decreases in PHQ-9 scores from baseline to 3 months and baseline to 6 months compared to TAU/DSS.

No significant decreases in PHQ-9 scores were observed in the subgroup analyses for any treatment arm when PHQ-9 was less than 10.

### Changes in EQ. 5D over time and between arms

There were no significant changes over time nor any significant differences between the groups for the EQ-5D.

### Dialysis attendance

There was no difference between groups in number of missed dialysis sessions at either 3 months or 6 months see Table 7.

**Table 7:** Average number of missing dialysis sessions (& 95% confidence Intervals) over the study follow-up dialysis

	0–3 months				*P* value comparing to ISS	4–6 months				*P* value comparing to ISS
	n	mean	95% CI			n	mean	95% CI		
ISS	60	4.3	3.5	5.1		55	3.6	2.8	4.4	
HepB/DSS	60	4.3	3.4	5.1	0.75	58	4.7	3.8	5.5	0.17
TAU/DSS	30	2.9	1.9	4.0	0.47	32	2.4	1.5	3.4	0.31

## Discussion

The HepB/DSS group showed decreased levels of distress and depressive symptoms at 3 months across the whole sample, with changes maintained at 6 months (after also receiving the Stay Strong app) although these changes did not attain clinical significance. When restricted to moderate/severely distressed or depressed participants (K10 > =25 or PHQ-9 > =10), participants in either the ISS or HepB/DSS groups demonstrated clinically significant changes in distress symptoms evident immediately for the HepB/DSS group (i.e. at 3 months) and at 6 months for both. Similar results were shown for depressive symptoms with significant changes at both 3 months and 6 months (which only approached clinical significance for the ISS group). Our findings of significant improvements in participants with more severe symptoms accord with previous studies examining effectiveness of apps for reducing depression. These have shown variation in outcomes based on symptom severity [25]. Future studies might therefore be more targeted by screening for symptom severity, as a floor effect will likely occur in participants with few symptoms: further improvement is both less likely and more difficult to detect in those who are already well.

As expected, baseline symptoms of depression and distress were relatively common in this group, with 45% (70/156) of participants scoring in the moderate/severe range on the PHQ-9 (i.e. > = 10) and 39% (61/156) scoring in the moderate/severe range on the K10 (i.e. > = 25). Rates of depressive symptoms were consistent with meta analyses previously reporting rates of 39% of dialysis patients experiencing depressive symptoms when assessed with an assessment tool [6]. However, previous research also suggests that cut off scores for assessment measures should be modified for those on dialysis given the overlap between somatic symptoms of depression and of dialysis itself [26].

Taken together, the results suggest that delivering culturally responsive app-based health assessments and interventions to Indigenous patients on dialysis can result in improvements in wellbeing. Given that depression is often underdiagnosed and undertreated in this group [26], this study suggests promising results can occur with relatively brief app-guided intervention. Brief, guided interventions such as those described here have potential to overcome some of the barriers to treatment described elsewhere, such as an already high medication burden, limiting willingness to add pharmacological treatments, or lack of motivation, resources or time for behavioural interventions [27]. Other reasons for low treatment rates include the lack of randomised controlled trial evidence demonstrating efficacy and safety of treatment regimens in CKD patients, confirming the importance of studies such as this one [26].

The Hep B app, which was chosen as a contact control, performed surprisingly well. It resulted in immediate change in distress and depressive symptoms (baseline to 3 months) which were maintained at 6 months after also receiving the Stay Strong App. On reflection, the Hep B Story was well aligned with participant identified needs and priorities, such as physical health and family. It was introduced as an intervention to promote physical health, with the goal at the end of the app being to share the information with family. It was also a less intense app to complete requiring a lower level of attention and concentration, and was available in one (top end) Indigenous language.

TAU/DSS appeared to show improvements in psychological distress (but not depressive symptoms) at 3 months for moderate/severely distressed participants. This change was not clinically significant and might be due to the small sample size in the sub analyses or related to spontaneous resolution of symptoms. The outcome assessments involved discussion of wellbeing and were delivered in local languages via an app. In consequence, it could be considered a digital mental health intervention. Assessment has previously been shown to have potential for a therapeutic effect, particularly when involving feedback [28]. Participants who scored highly on the K10 received follow up questions (per risk protocol) and were referred. Participants commented on the assessment app during the study stating, “It’s good. No one has ever talked to me about this before”.

The addition of a Stay Strong intervention at 3 months for those who had received the Hep B Story at baseline did not result in further improvement, but the gains made were maintained. Lack of improvement from 3 to 6 months may be due to significant improvements observed already in both HepB/DSS and TAU/DSS groups from baseline to 3 months, and thus no room for further significant improvement. It is unclear whether differences between the groups might have been observed at longer follow-up periods (e.g. 12 months), after participants had had a chance to practice the skills gained through receiving the Stay Strong intervention (and having these reinforced a second time for the intervention group). However, a 12-month follow up was considered difficult in this population given the high morbidity and mortality. Nine participants died during the study period, five of whom were in the Stay Strong intervention group.

Further limitations included the small sample size, particularly for sub-analyses, and the relatively short follow up period. Hence, findings should be interpreted with caution. Lack of a contact control group without the use of an app might also be considered a limitation.

No changes in EQ-5D scores were observed for this group and the utility of this measure in this setting is unclear. This measure was included as a quality of life measure to contribute to the cost-effectiveness analysis (publication in preparation) in order to calculate QALYs, however researcher experience of its use suggests its ability to tap into the quality of life domains valued by this population may be limited [29, 30]. Similarly, no significant differences were identified between the groups on number of missed dialysis sessions, suggesting no impact of the interventions on treatment adherence. While literature suggests depression treatment can improve depressive symptoms in people with kidney disease, it is still unclear whether such therapy improves physical health outcomes [31].

More research is required to understand the specific active components of the interventions [32]. One potential active component is clinician guidance. Previous literature suggests that effect sizes of treatment apps can be influenced by clinician or other guidance, with guided interventions demonstrating a larger effect on outcomes than unguided interventions [33]. Both active conditions (Stay Strong app and Hep B app) were clinician-guided, which may have contributed to our results.

While the interventions in this study were developed specifically for Indigenous Australians, the findings have relevance for other first nations or minority populations internationally [34, 35]. Other first nations groups share the heavy burden of ESKD and its associated challenges including separation from country and family, cultural isolation, uncertainty around accommodation and establishing life in an urban context, similarly impacting perceived quality of life [36]. Furthermore, divergent health beliefs between health care providers and minority groups are identified as challenges to delivery of effective health care with culturally appropriate communication strategies needed to address this issue [36]. This study showed that culturally adapted digital mental health interventions can be acceptable and effective. Meaningful user involvement in the development of mobile health technologies has been found to increase engagement and acceptability however, evidence for effectiveness remains limited [34]. The Framework for the Development and Evaluation of RCTs for Complex Interventions to Improve Health recognises there are specific difficulties in testing complex interventions and recognises that complex interventions may work best if they are tailored to local contexts [32]. Although acceptability and engagement are commonly assessed, and greater attention to early phase piloting and development work has been recommended, a focus on high quality RCTs such as this one is also needed to improve access to evidence-based, effective interventions to improve wellbeing and quality of life for first nations and other minority groups internationally.

## Conclusion

This is the first RCT to examine efficacy of a dMH tool in an Indigenous CKD setting. Findings suggest that using apps for treatment can improve wellbeing for people on dialysis. Wellbeing apps like Stay Strong may work best for those who already have symptoms of distress or depression, and when delivered more than once. Simple, brief apps that focus on physical health through storytelling may also lead to important improvements in wellbeing. Further research is required to replicate these findings and identify active intervention components.

## Data Availability

The datasets generated and/or analysed during the current study are not publicly available due to participant confidentiality but may be available from the corresponding author on reasonable request.
